# Periostin as a Biomarker in the Setting of Glomerular Diseases—A Review of the Current Literature

**DOI:** 10.3390/biomedicines10123211

**Published:** 2022-12-10

**Authors:** Nicolae Pană, Cristina Căpușă

**Affiliations:** 1 Faculty of Medicine, Carol Davila University of Medicine and Pharmacy, 050474 Bucharest, Romania; 2 Diaverum Morarilor Clinic of Nephrology and Dialysis, 022452 Bucharest, Romania; 3 “Dr Carol Davila” Teaching Hospital of Nephrology, 010731 Bucharest, Romania

**Keywords:** periostin, biomarker, glomerular disease

## Abstract

Chronic kidney disease (CKD) is a highly prevalent and potential progressive condition with life-threatening consequences. Glomerular diseases (glomerulopathies) are causes of CKD that are potentially amenable by specific therapies. Significant resources have been invested in the identification of novel biomarkers of CKD progression and new targets for treatment. By using experimental models of kidney diseases, periostin has been identified amongst the most represented matricellular proteins that are commonly involved in the inflammation and fibrosis that characterize progressive kidney diseases. Periostin is highly expressed during organogenesis, with scarce expression in mature healthy tissues, but it is upregulated in multiple disease settings characterized by tissue injury and remodeling. Periostin was the most highly expressed matriceal protein in both animal models and in patients with glomerulopathies. Given that periostin is readily secreted from injury sites, and the variations in its humoral levels compared to the normal state were easily detectable, its potential role as a biomarker is suggested. Moreover, periostin expression was correlated with the degree of histological damage and with kidney function decline in patients with CKD secondary to both inflammatory (IgA nephropathy) and non-inflammatory (membranous nephropathy) glomerulopathies, while also displaying variability secondary to treatment response. The scope of this review is to summarize the existing evidence that supports the role of periostin as a novel biomarker in glomerulopathies.

## 1. Introduction

Periostin is a 90 kDa non-structural matricellular protein originally described in osteoblastic cells lineages, hence the reason it was initially referred to as OSF-2—osteoblast stimulating factor 2 [1,2,3]. It is highly expressed in the embrionary periosteum, in the periodontal ligaments, and during the processes associated with organogenesis, normally acting as a cell-adhesion molecule for preosteoblasts, participating in osteoblast recruitment and spreading [1,2,4]. Along with other matricellular non-structural proteins (not involved in the structural architecture of the tissue, e.g., thrombospondins, tenascins, osteonectin, or osteopontin), periostin expresses binding domains to cellular receptors (integrins), to the extracellular matrix, and to growth factors, through which it is involved in local processes of adhesion, migration, proliferation, and cellular differentiation through its ability to transduce and modulate transcellular signaling [3,4].

The periostin molecule comprises three distinct regions with different purposes [4,5,6]:

- A C-terminal domain which is a site of proteolytic cleavage that renders the different isoforms of periostin;
- An N-terminal EMI domain which renders the capacity to interact with collagen I and fibronectin;
- A middle region consisting of four fasciclin-I domains that contain binding sites for bone morphogenetic protein 1 (BMP-1) and α_V_β_3_ and α_V_β_5_ integrins, through which it manifests its aforementioned capacity to mediate cell adhesion and migration.

This palette of sites for interaction with extracellular matrix (ECM) components and cell-surface receptors provides periostin with unique capabilities, to modulate both the architectural properties of the surrounding connective tissue and the cell–matrix interactions [4].

Thus, periostin plays an important role in tissue regeneration, remodeling, and fibrosis [7]. In addition, a growing body of evidence links periostin to inflammatory states and tumorigenesis, as abnormal periostin secretion could contribute to aberrant remodeling and homeostasis of the tissue microenvironment [1,7]. In the heart, periostin expression correlated with myocardial healing in the post-myocardial infarction period and periostin-null mice exhibited a lower degree of local fibrosis and hypertrophy [8,9]. Periostin seems to play an important role in lung diseases, as reports on idiopathic pulmonary fibrosis and asthma refer to both its prognostic potential and its mediatory role in disease development [10,11]. Clinical studies supported the ability of serum periostin levels to reflect asthma response to therapy [12,13], as it has been demonstrated that periostin is an intermediate step in a cascade process that involves stimulatory signals from IL13 and IL4 for periostin expression, which subsequently facilitates Th2 inflammatory response [14]. However, in later phase 3 randomized control trials, LAVOLTA I and LAVOLTA II, the anti-IL13 monoclonal antibody lebrikizumab, reduced asthma exacerbations in periostin-high subjects, but the effect reached statistical significance only in the former study, despite the inhibition of IL13 axis in both, which likely points to the complexity of cell–matrix interactions and loopholes through which inflammation and fibrosis ultimately develop [15]. As mentioned, periostin involvement in tumorigenesis and metastasis is well documented [16,17], as tissular expression was positively correlated with a poor survival prognosis in renal cell carcinoma patients [18]. High tissular expression of periostin was also reported in skin lesions from systemic scleroderma or atopic dermatitis [1,4].

## 2. Periostin Functions and Mechanisms

The multiple fibrotic and inflammatory mechanisms through which periostin expression is stimulated and thereafter exerts its actions have been described and are subject to further update.

There is proof that angiotensin-II (AT-II) stimulates periostin expression in myocardial fibroblasts and smooth muscle cells through the phosphatidil-inositol-3 kinase (PI-3 kinase), Ras/p38 MAPK/CREB, and ERK1/2/TGF-β1 pathways [19,20], as increased periostin levels in rat fibroblasts have been observed in response to AT-II infusion [20]. This relation seems to be bidirectional as periostin downregulation in a 5/6-nephrectomy model led to a downregulation of RAAS-activation [21]. Furthermore, in the previously described hypertensive nephropathy murine model by Guerrot et al., treatment with losartan reduced the expression of periostin in renal tissue and proteinuria and improved kidney hemodynamics [22]

Another well-described relationship is that with TGF-β1, which also seems to act in both directions: on one hand, TGF-β1 induces periostin expression and facilitates the transition to a mesenchymal phenotype for the tubular renal cells by de novo expression of mesenchymal cell phenotypes markers (vimentin, N-cadherin), loss of epithelial cell markers (E-cadherin), and disruption of intercellular junctions (tight junction, adherens junction) [23,24]; on the other hand, periostin promotes TGF-β release from immune cells and enhances adhesion [25]. Similar findings have been replicated in human biopsy specimens, as in vitro stimulation of mesangial cells by TGF-β1 infusion led to significant periostin expression with downstream cellular proliferation and inhibition of the caspase-3 apoptotic mechanism [26].

There is also compelling evidence from Prakoura et al. that periostin induced by the nuclear factor kappa-light-chain-enhancer of activated B cells (NF-κB) promotes renal injury in a glomerular disease model [27]. Platelet-derived growth factor B (PDGF-B) has also been proven to promote periostin expression in the mesangium with downstream cellular proliferation and ECM expansion [28,29]

The mechanisms involved in the downstream effects of periostin are multiple and constantly updated, as it does not only trigger specific paths, but often, as previously described, provides positive autocrine-like feedback loops that enhance the expression of its inducers. It binds to cell-surface integrin receptors integrin αVβ3 and αVβ5 and throughout the activation of the PI-3 kinase mediates migration, differentiation, and adhesion of tumoral, vascular smooth muscle, and mesenchymal stem cells [29]. Periostin seems to exert a more direct role in fibrosis development, by interacting with collagen and other ECM components in order to facilitate cross-linking and the inclusion of the former in the fibrotic mass [29,30]. Periostin can also bind to integrin cell-surface receptors and mediate cell apoptosis through the integrin-linked kinase (ILK) [31], as it may also induce integrin-β3 expression and consequently trigger its inflammatory pathway [32]. There are supporting data that periostin may either directly act on the aforementioned pathways, or also use intermediate routes, as in the case of PPARα, in which periostin decreases PPARα expression which would normally inhibit the TGF-β/Smad3 signaling route, therefore providing an indirect stimulus for ECM expansion [33,34].

**Figure 1:**
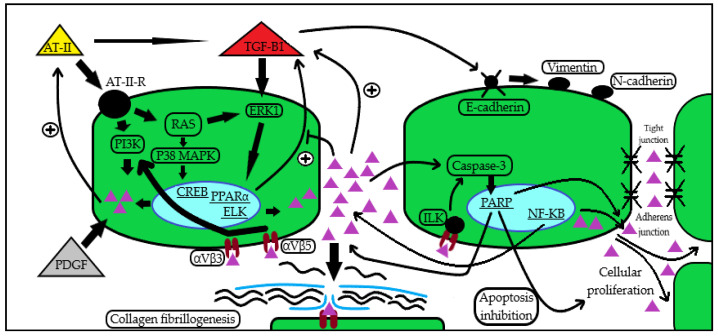
Periostin induction and action pathways in kidney disease. Periostin seems to be induced by a series of cytokines and growth factors (AT-II [19,20], TGF-β1 [23,24], NF-κB [27], PDGF [28,29]), subsequently leading to the activation of signaling pathways [19,20,23,25,27,28,29] that mediate ECM assembly through collagen fibrillogenesis [29,30], promotion of pro-inflammatory and anti-apoptotic routes [25,26,31], epithelial–mesenchymal transition with cellular migration, and enhanced adhesion and proliferation [23,24,31,32]. In the figure, the periostin molecule is illustrated by the all-purple triangular shape. Abbreviations: AT-II: angiotensin II; AT-II-R: angiotensin II receptor; RAS: Rat sarcoma virus GTP-ase; TGF-β1: transforming growth factor β1; NF-κB: nuclear factor kappa-light-chain-enhancer of activated B cells; PDGF: platelet-derived growth factor; ECM: extracellular matrix; PI3K: phosphatidyl inositol 3-kinase; ERK1: extracellular signal-regulated kinase 1; P38 MAPK: P38 mitogen-activated protein kinase; CREB: cAMP-response element-binding protein; PPARα: peroxisome proliferator-activated receptor alpha; ELK: ETS-like protein; ILK: integrin-linked kinase; PARP: poly ADP-ribose polymerase; αVβ3: αVβ3 integrin; αVβ5: αVβ5 integrin.

A more illustrative take on the potential mechanisms of periostin action in kidney disease can be seen in Figure 1.

## 3. Periostin in CKD and in the Kidney

Chronic kidney disease (CKD), an increasing public health problem with a prevalence of 10–13% worldwide [35], can be described as a potential progressive condition due to various initial kidney injuries which trigger inadequate repair mechanisms, finally leading to nephron loss and to deleterious compensatory hyperfunction of the remnant nephrons [36]. Since progressive kidney diseases are characterized by ECM deposition in glomeruli and interstitia, vascular rarefaction, inflammatory cell infiltrate, and nephron loss [37], it is conceivable that matricellular proteins are involved in the pathophysiology of these conditions and their expression and urinary levels could be of interest as disease biomarkers. Currently, the usefulness of existing tissue and urine markers to predict outcomes in CKD is not optimal [38]. Therefore, it is of paramount importance to identify molecules that add value to clinical predictors of treatment response and prognosis in patients with kidney diseases. 

In the renal biopsies of healthy kidney donors, periostin was constantly expressed in the vascular pole of the glomerulus and around Bowman’s capsule, and lacking in the tubular compartment [26], while there are also reports that describe a complete absence of periostin in the non-pathological states [39]. 

During the last two decades, increasing evidence has unraveled the involvement of periostin in CKD. Various experimental models revealed an increased expression of periostin, including in the 5/6 nephrectomy, unilateral ureteral obstruction, and streptozocin-induced diabetic nephropathy [23,24,33]. In the UUO model, initial periostin staining was observed in the collecting tubules and subsequently in the distal and proximal tubules, a finding consistent with this disease’s pattern of progression [23]. Additionally, in a murine model of L-NAME-induced hypertensive nephropathy, periostin was amongst the highest expressed in the transcriptomic analysis, and its magnitude correlated with disease progression and with the kidney function and damage markers—serum creatinine, proteinuria, and renal blood flow [22]. 

**Figure 2:**
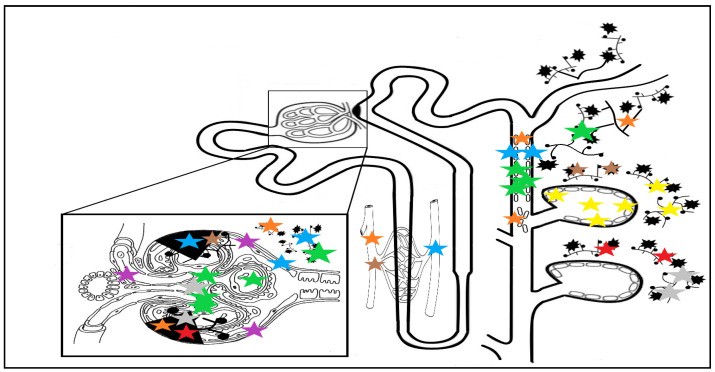
Nephronal sites of periostin staining depending on underlying disease: yellow stars indicate staining in ADPKD in cyst fluid, ECM and epithelial cyst cells [42]; blue stars indicate impregnation in DN in glomerulosclerosis areas, periglomerular fibrosis and ECM, atrophic tubules, around fibrotic vessels, and in Bowman’s capsule [24,40]; green stars indicate IgAN staining: in all nephronal sites, especially in the mesangium, periglomerular fibrosis, tubular cells, and casts and interstitial fibrosis; grey and red stars indicate MN and FSGS staining, occurring in fibrotic and glomerulosclerosis areas, with staining in the mesangium only for MN [26]; a similar staining pattern with a more pronounced expression in the glomerular and interstitial vascular compartments can be seen in hypertensive nephropathy, in the brown stars topography [22]; staining in lupus nephritis patients (orange stars) occurs in fibrotic vessels, tubular epithelial cells and tubular cells casts, in areas of interstitial fibrosis, and in the sclerotic glomerular and periglomerular sites [41]. The purple stars indicate staining in the nephrons of patients without kidney disease, with minimal vascular pole and Bowman capsule staining, sparing the tubulo-interstitium [26]. Abbreviations: ADPKD: autosomal dominant polycystic kidney disease; ECM: extracellular matrix; DN: diabetic nephropathy; IgAN: IgA nephropathy; MN: membranous nephropathy; FSGS: focal segmental glomerulosclerosis.

Similarly, a significant increase in the expression of periostin in renal tissue during progressive kidney injury was also observed in adult humans, with specific topography depending on the underlying injury: in areas of glomerular sclerosis, Bowman capsule, ischemic lesions and tubular cytoplasm in diabetic subjects [40]; in areas of interstitial fibrosis, fibrotic vessels, in the atrophic tubules and in renal tubular epithelial cells in lupus nephritis [41]; and in the cystic fluid, cyst extracellular matrix, and epithelial lining cells of the cysts in autosomal dominant polycystic kidney disease (ADPKD) [42]. The specific sites of periostin staining in kidney diseases are illustrated in Figure 2.

On the other hand, mice lacking periostin gene expression presented with a more attenuated interstitial fibrosis and were protected against CKD progression [23]. In vivo delivery of antisense oligonucleotides inhibiting periostin expression protected the murine subject against the L-NAME-induced renal injury. This seems to be secondary to TGF-β stimulation since the renal epithelial cell seems to lose its epithelial phenotypes after in vitro administration of this [23].

## 4. Periostin as a Biomarker in Glomerular Disease

Glomerular diseases are conditions that comprise a distinct subset of CKD causes that are potentially amenable by specific therapy. Bearing in mind their individual specific pathways for development and progression, they are all characterized by local inflammation and activation of pro-fibrotic mechanisms. In order to assess the efficacy of treatment and prognosis in glomerular diseases, novel biomarkers are needed, since currently kidney injury is monitored in clinical practice only through markers that measure kidney functional loss—i.e., serum creatinine, urine protein, albumin, and urine cystatin-C—but not kidney cellular injury. These newly proposed markers of kidney function loss may have the advantage of recognizing kidney injury before the onset of proteinuria or before the decline in eGFR, therefore providing an opportunity for early nephroprotection. Some other urinary proteins are also markers of tubulointerstitial injury but are not routinely measured or used in clinical practice (NGAL, KIM-1, IL-18).

A 2021 paper by Chimenz et al. highlights the potential of other ECM molecules to serve as biomarkers of glomerular disease, as high mobility group box 1 (HMGB1) and TGFβ-1 were able to predict subclinical fibro-inflammatory changes in the kidney by their early detection in the serum and urine of Alport disease subjects as compared to healthy subjects before the onset of proteinuria. HMGB1 appears to play several roles in cellular signaling and pro-inflammatory pathways, and several studies have reported elevated serum and urine levels in lupus nephritis and in anti-neutrophil cytoplasmic antibody (ANCA)-associated vasculitis with renal involvement. TGFβ-1 seems to be directly upregulated by HMGB1-triggered routes, and there is supporting evidence of elevated urinary levels of TGFβ-1 in children with idiopathic nephrotic syndrome and in focal and segmental glomerulosclerosis (FSGS) [43]. In order for a biomolecule to be utilized as a biomarker, it must first meet a series of universal characteristics: it has to be non-invasive by having a readily available source (blood, urine), easily measurable, inexpensive, and able to provide rapid day-by-day results; it should also have sensitivity in order to provide early detection and no overlap with healthy individuals, high specificity in order to be up/downregulated in the target population and uninfluenced in comorbid conditions, and their levels should vary in response to treatment in order for them to have a prognostic significance [44]. 

Periostin has already proven its usefulness as a biomarker in fibrotic diseases and in cancer states [25,45], owing this to some of its features: it is increased in many pathological states in the urine, blood, and sputum, thus proving that is readily secreted from injury sites, the concentration of serum periostin revolves around the ng/mL domain (≈10 ng/mL), an appropriate magnitude for a precise detection of variations in the investigated site [13,46]. A potential disadvantage could be posed by the fact that inflammatory based comorbid conditions could confound humoral levels when present, impairing periostin level interpretation. Another potential challenge that limits its use in the under 5-year-old pediatric population is that basal serum periostin levels are elevated as a consequence of bone growth activity [47]. Periostin can also be subject to serum level variations in physiological states: body mass index seems to be negatively correlated with serum levels, a circadian rhythm analysis indicates higher levels in the morning, while gender reports do not consistently indicate a significant difference in this instance [46].

Periostin checks several criteria of biomarkers: its expression is minimal in healthy subjects, while it is highly induced in several kidney disease models (UUO, hypertensive nephropathy, 5/6 nephrectomy, ADPKD) and its local magnitude of expression correlates with kidney function decline and histological involvement [22,23,27,42]. A study aiming to describe expressed protein transcripts in patients with glomerular diseases identified periostin as the most expressed matricellular protein [26].

Similar to the blood fraction, the urinary fraction of periostin shares a magnitude of less than 1 ng/mg creatinine, but this fraction can significantly increase and there is evidence that points towards a positive correlation with disease severity in multiple CKD causes [4,40,41].

The urinary fraction of periostin seems to originate from the tubulointerstitium since serum periostin levels have not shown a reliable prognostic value in kidney disease and urinary periostin levels have not been significantly different between proteinuric and non-proteinuric conditions [24,48].

As previously mentioned, glomerular diseases are causes of CKD that are potentially amenable to specific ethiopathogenic treatments that are proven to impact long term prognosis. Glomerulopathies can be divided in inflammatory and non-inflammatory diseases, depending on the main pathogenic mechanism that drives the initial injury: conditions like IgA nephropathy are characterized by the mesangial cellular proliferation with local inflammation and ECM expansion secondary to local immunoglobulin complex deposition, whereas diseases like membranous nephropathy or focal segmental glomerulosclerosis are mainly characterized by direct podocyte injury that initially lack overt histological inflammatory features.

Of all these glomerulopathies, IgA nephropathy (IgAN) is the most incident and prevalent and is a leading cause of CKD, being the most common glomerular disease in adults, with an overall incidence of 2.5 in 100,000 individuals as predicted by biopsy registry data; this in fact might underestimate the true disease burden due to selection bias imposed by kidney biopsy indications [49]. In addition, a detailed, structured, and standardized pathological scoring system was established for IgAN [50], making it a favorable clinical setting to investigate the potential involvement of periostin as a prognostic biomarker. To date, only a few studies have addressed the issue of periostin with clinical and pathological findings in IgAN. Firstly, Hwang et al. found that baseline urinary excretion of periostin correlated with renal fibrosis and predicted renal outcomes in patients with IgAaN, with a moderate diagnostic profile of urinary periostin-to-creatinine ratio (AUC: 0.782) for predicting CKD progression in IgAN [51]. Secondly, Wantanasiri et al. reported a worse kidney function in an IgAN and lupus nephritis small cohort that had a higher urinary periostin excretion compared to healthy controls; they also reported on the significantly lower urinary periostin levels at 6 months of follow-up in patients with response to treatment [52]. The same research group reported on the immunohistochemical staining of periostin in an IgAN subject population and found periostin staining in glomerular, tubulointerstitial, and vascular compartments, with periglomerular staining being positively correlated with interstitial fibrosis, as was the case for tubular cell cast staining and tubular epithelial cell staining in relation with tubular atrophy staining [53]. The Polish research group of Mizerska-Wasiak et al. failed to replicate the previously mentioned results of Hwang et al. [51], but their analysis of a 20 patient cohort with IgAN may have been confounded by the fact that the analysis was performed in a pediatric population (3 to 17 year old subjects), by the lack of measurement of urinary periostin at baseline (kidney biopsy moment), and by the fact that the subjects had low severity CKD stage G1 to G2 [54]. New data support the fact that periostin acts directly on the mesangial cell, as exogenous administration of periostin led to an increase in proliferating cell nuclear antigen (PCNA) with downstream enhancing of mesangial cell proliferation; its expression in kidney tissue was also correlated with serum creatinine levels and eGFR [55].

Lupus nephritis (LN) is the second most common causes of nephritic syndrome in the general population [56] and a gene profiling data analysis identified the periostin expression gene as one of four extracellular protein differentially-expressed genes in this setting [57]. The same research group of Wantanasiri et al., in a study on 42 kidney biopsies from patients with lupus nephritis, reported that the periostin staining score was directly associated with the histopathological chronicity index and, accordingly, with worsening renal outcomes [41]. Similar findings were described for the urinary fraction of periostin in the setting of lupus nephritis [52]. The research group of Sen et al. [26] describe periostin staining in the glomerular tuft and in periglomerular capillaries, as well as in the tubulointerstitial compartment, the only nephronal region that seems to be spared in healthy controls. The quantitative report of staining in LN subjects was significantly higher in the lower eGFR subjects. Of note, one subject presented with mild periostin staining in the tubulointerstitium, although he presented with an eGFR above 60 mL/min at baseline. Additionally, the mesangial positivity of staining was increased with disease vintage in a LN subject with serial kidney biopsies [26].

Membranous nephropathy (MN) is the leading cause of nephrotic syndrome in the adult non-diabetic population and the identification of PLA2R antigen involvement in primary MN has led to a paradigm shift in terms of potential non-invasive diagnosis and an efficient prognostic monitoring of disease response through the anti-PLA2R antibodies [58]. The transcriptomic analysis by Sen et al. [26] describes periostin as the most expressed matricellular protein in MN, as these proteins were highly expressed on progressive proteinuric glomerular diseases, such as focal segmental glomerular sclerosis (FSGS) and MN, and relatively low or absent in non-progressive nephropathies, such as minimal change disease (MCD). Real-time PCR for the confirmation of the micro-array analysis was performed and, although statistical significance was not reached, a trend for induction in MN was observed, and similar results were obtained for the kidney biopsies of patients with IgAN. Periostin staining was reported in areas of interstitial inflammation and fibrosis in the tubulointerstitium of patients with glomerular diseases (including MN) and kidney functional decline, independently of the type of glomerular disease. A quantitative periostin staining analysis found a positive correlation with worsening kidney function in these subjects. Impregnation was mostly observed in areas with ECM accumulation and tubular atrophy. Additionally, since podocyte injury is central to primary MN, its assessment through quantification of urinary podocyte shedding seems to be associated with disease activity markers (proteinuria and anti-PLA2R antibody titers), although in a non-linear manner [59]. Another 2013 study analyzed the urinary shedding of podocyte mRNA pellets in primary MN and reported on the positive association of proteinuria and podocyturia, although in a less strong fashion than in the case of MCD [60]. Since the urinary fraction of periostin seems to be derived from the tubulointerstitium (as previously mentioned [24,48]) and podocyte depletion leads to mesangial expansion, secondary FSGS and then global sclerosis (when depletion is above 70%) [61], it is reasonable to assume that a simultaneous detection of urinary periostin and podocyturia can provide relevant information on the evolutive continuum of primary MN and guide management, since the onset of periostin excretion could suggest the establishment of fibrosis in the tubulointerstitium, even when the disease is in remission (no active podocyturia). Data seem to be lacking regarding secondary causes of MN. 

Similar findings are also reported in the analysis of Sen et al. [26] for the kidney biopsies from subject with FSGS, although minimal mesangial impregnation was a distinct finding, with important periostin representation only in sclerotic interstitial areas. As previously mentioned, the transcriptomic analysis and thereafter the microarray found minimal representation of periostin in the biopsies of MCD subjects, a finding consistent with the long-term evolution of MCD that is rarely of long-term kidney functional decline [26]. Additionally, Sen et al., or other study groups, seem to make no reference to the primary, secondary, or genetic origin of FSGS [26]. We can hypothesize that periostin may be equally involved in the vast majority of secondary FSGS cases characterized by glomerulosclerosis through hyperfiltration on a low remnant nephron mass, as this is one of the main mechanisms through which the already described model of hypertensive nephropathy progressed to end stage kidney disease [22].

Regarding kidney allograft dysfunction, antibody-mediated rejection (ABMR) is currently the leading cause of kidney allograft failure [62], with histologic evidence of glomerulitis and peritubular capillaritis accounting for active ABMR and with transplant glomerulopathy features indicating chronic active ABMR, which impacts long-term prognosis and treatment [63]. This is important because Th2 lymphocyte response is central to the pathogenesis of ABMR, and we highlighted the implication of periostin in the Th2 axis [14]. A 2022 proteomic study revealed periostin as one of the highest correlated proteins with chronic glomerular injury and amongst the highest expressed molecules in subjects with histological proof of chronic active ABMR compared to those with active ABMR (3.8-fold change) [63], highlighting its prognostic value and the potential to avoid immunosuppression in cases that display irreversible histological features. A 2014 urinary periostin report indicates that kidney allograft dysfunction subjects had a statistically significant higher urinary fraction than transplant patients with normal kidney function, and that this fraction correlated directly with urine protein-to-creatinine ratio and serum creatinine. Satirapoj et al. also reported a good diagnostic profile of urinary periostin-to-creatinine ratio (AUC: 0.830) for detecting renal fibrosis in patients with kidney allograft dysfunction [39].

Early detection is also mandatory in the early stages of diabetic nephropathy (DN), before the onset of albuminuria. Satirapoj et al. [40] evaluated urinary excretion and tissue impregnation of periostin in a diabetic cohort comprising both normoalbuminuric and subjects with incremental levels of albuminuria and reported on the elevated urinary periostin in both groups, which correlated with kidney functional decline, aging, and albuminuria magnitude. The author found that urinary periostin levels increase before the onset of albuminuria and that urinary periostin may act as an early biomarker of renal cell injury in type 2 diabetic patients without albuminuria [40].

## 5. Clinical Implications

As previously mentioned, other molecules belonging to the ECM domain have been described not only as elevated in multiple kidney disease states with glomerular involvement (HMGB1 in LN and in ANCA-associated vasculitis, TGFβ-1 in FSGS and in DN), but as potentially upregulated before the onset of proteinuria and overt kidney functional decline as evaluated by eGFR [43]. Future prospects on an earlier diagnosis and more efficient management of glomerulopathies may include the development of combined panel assays of these biomarkers, as they may display different and partial overlapping characteristics that may better suit simultaneous quantification. As some markers may prove to be more specific and less sensitive, simultaneous determination with other markers that may display more early detection but in a less specific manner (e.g., potentially influenced by comorbid states), could potentially provide a wider array of information that, when integrated in clinical context, could aid in attaining more efficient nephroprotection.

One of the mainstays of periostin’s prognostic value, as described in the case of kidney allograft dysfunction, could be that of differentiation between chronic active and chronic histological lesions in the kidneys of glomerulopathic patients, with subsequent impact on immunosuppression prescription.

These individual biomarkers can be combined in assays that can thereafter be tested and validated in multiple diseases, including glomerulopathies, as similar combined detections of urinary markers are already reported in settings like acute kidney injury (AKI) [64], with promising early results.

Favorable prospects are also available in regard to the potential blockade of the periostin axis through specific delivery of antisense oligonucleotides, as Mael-Ainin et al. proved in their 2014 murine models [23]. Similar data have been replicated more recently by Tomaru et al. in an idiopathic pulmonary fibrosis model [65], and by Kobayashi et al. in a model of non-alcoholic steatohepatitis [66].

Although these data are encouraging regarding the potential role of periostin in kidney and glomerular diseases, one must bear in mind that the molecular pathways presented are intertwined and “escape routes” of fibro-inflammatory mechanisms are not uncommon, as proven in the past by the fact that basic science data cannot be fully translated and replicated in vivo and in real-life settings.

## 6. Conclusions

In conclusion, periostin seems to bear multiple features that could support its role in the future for the prognostic evaluation of patients with glomerular disease, as reports in murine disease models and in human biopsies have highlighted the minimal expression in healthy individual and the enhancement of expression in disease models, although further prospective evaluation is necessary to support its prognostic utility. Furthermore, the forever expanding field of novel molecular biomarkers could potentially lead to standardized and potentially combined (with other biomarkers) assays for urine and blood samples in order to construct and validate large-scale prognostic models.

Besides the aim of suggesting that periostin has prognostic potential in glomerulopathies, the review also intends to bridge the gap between basic science and clinical medicine, by covering available data in both fields in a translational manner, thus providing rationale for further clinical research on the roles of periostin.

## Data Availability

Not applicable.
